# Supplementary material for: The Disparities in Mental Health Between Gay and Bisexual Men Following Positive HIV Diagnosis in China: A One-Year Follow-Up Study
Source: Int J Environ Res Public Health. 2020 May 14;17(10):3414. doi: 10.3390/ijerph17103414 (PMC7277388; doi:10.3390/ijerph17103414)
Supplement: Supplementary file 1 [file ijerph-17-03414-s001.pdf]

Table S1. Differences in baseline sample characteristics between participants who completed follow-up survey and those who did not.

Characteristics	Gay Men (*n* = 235)		*p*	Bisexual Men (*n* = 119)		*p*
	Complete (*n* = 171)	Loss (*n* = 64)		Complete (*n* = 87)	Loss (*n* = 32)	
Age						
18–29	115 (67.3%)	45 (70.3%)	0.654 [1]	60 (69.0%)	18 (56.3%)	0.196 [1]
≥ 30	56 (32.7%)	19 (29.7%)		27 (31.0%)	14 (43.8%)	
Marital						
Single	141 (82.5%)	51 (79.7%)	0.861 [1]	62 (71.3%)	21 (65.6%)	0.638 [1]
Married	18 (10.5%)	6 (9.4%)		19 (21.8%)	8 (25%)	
Divorce	12 (7.0%)	7 (10.9%)		6 (6.9%)	3 (9.4%)	
Education background						
Senior or lower	70 (40.9%)	27 (42.2%)	0.862 [1]	38 (43.7%)	18 (56.3%)	0.223 [1]
College or higher	101 (59.1%)	37 (57.8%)		49 (56.3%)	14 (43.7%)	
Employment						
Employed	111 (64.9%)	38 (59.4%)	0.466 [1]	57 (65.5%)	27 (84.4%)	0.058 [1]
Unemployed	60 (35.1%)	26 (40.6%)		30 (34.5%)	5 (15.6%)	
Monthly income (RMB)						
≤ 4000	96 (56.1%)	35 (54.7%)	0.967 [1]	54(62.1%)	14 (43.8%)	0.094 [1]
> 4000	75 (43.9%)	29 (45.3%)		33 (37.9%)	18 (56.2%)	
Children						
With	21 (12.3%)	10 (15.6%)	0.500 [1]	21 (24.1%)	8 (25.0%)	0.923 [1]
Without	150 (87.7%)	54 (84.4%)		66 (75.9%)	24 (75.0%)	
Living alone						
Yes	46 (26.9%)	27 (42.2%)	**0.024** [1]	29 (33.3%)	8 (25.0%)	0.384 [1]
No	125 (73.1%)	37 (57.8%)		58 (66.7%)	24 (75.0%)	
HIV-related symptoms						
With	61 (35.7%)	19 (29.7%)	0.389 [1]	37 (42.5%)	8 (25.0%)	0.080 [1]
Without	110 (64.3%)	45 (70.3%)		50 (57.5%)	24 (75.0%)	
CD4 count (cells/mm)						
≤ 350	66 (38.6%)	28 (44.4%)	0.418 [1]	40 (46.0%)	13 (40.6%)	0.602 [1]
> 350	105 (61.4%)	35 (55.6%)		47 (54.0%)	19 (59.4%)	
PHQ-9	8 (4, 13)	6 (2, 10)	0.305 [2]	9 (4, 15)	10 (5, 16)	0.829 [2]
GAD-7	6 (3, 10)	6 (2, 10)	0.729 [2]	7 (4, 13)	7 (4, 14)	0.712 [2]
HIV/AIDS-related stress	21 (14, 30)	21 (16, 30)	0.894 [2]	24 (13, 35)	22 (11, 34)	0.907 [2]
Social stress	12 (7, 16)	13 (8, 16)	0.928 [2]	12 (8, 16)	12 (9, 15)	0.685 [2]
Emotional stress	6 (3, 10)	5 (2, 8)	0.393 [2]	6 (3, 12)	6 (3, 11)	0.755 [2]
Instrumental stress	4 (1, 7)	4 (1, 7)	0.820 [2]	5 (2, 8)	4 (2, 8)	0.808 [2]
Social support	28 (23, 33)	29 (21, 33)	0.627 [2]	28 (23, 32)	32 (24, 35)	0.218 [2]
Objective support	13 (10, 17)	14 (10, 19)	0.285 [2]	14 (11, 19)	14 (10, 20)	0.556 [2]
Subjective support	8 (6, 10)	7 (5, 9)	0.996 [2]	8 (5, 9)	8 (7, 9)	0.464 [2]
Support utilization	6 (5, 7)	6 (5, 8)	0.843 [2]	6 (5, 7)	6 (5, 8)	0.657 [2]

[1] Chi-square tests; [2] Mann-Whitney U tests.
